# Effects of COVID-19 on the Sustainable Development Goals (SDGs)

**DOI:** 10.1007/s43621-021-00026-x

**Published:** 2021-03-17

**Authors:** Kalterina Shulla, Bernd-Friedrich Voigt, Stefan Cibian, Giuseppe Scandone, Edna Martinez, Filip Nelkovski, Pourya Salehi

**Affiliations:** 1 ZEF-Center for Development Research, University of Bonn, Bonn, Germany; 2 Institute for Personnel and Organizational Research (Ipo), FOM–University of Applied Sciences, Münster, Germany; 3 Center on Global Affairs and Postdevelopment (C-GAP), Făgăraș Research Institute (FRI), Făgăraș, Romania; 4 QG Enviro, Lecce, Italy; 5 Proactivo Sostenible, Mexico City, Mexico; 6 UNDP, Skopje, Republic of North Macedonia; 7 ICLEI World Secretariat, Bonn, Germany

**Keywords:** Sustainable Development Goals, COVID-19 pandemic, Interdependencies

## Abstract

Global crises caused by the pandemic of COVID-19, since early 2020, can compromise the world commitment to the 2030 Agenda for Sustainable Development. This study discusses critical aspects of the global pandemic for the achievement of the Sustainable Development Goals (SDGs). More precisely, we analyze how the new circumstances created by the pandemic have affected the interdependencies between SDGs. Following a synopsis of the current literature, we focus on effects regarding SDG3 (Health & Well-Being), SDG4 (Quality Education), SDG8 (Decent Work & Economic Growth), SDG12 (Consumption & Production) and SDG13 (Climate Action). Following a qualitative research approach, we based our analysis on moderated focus group discussions (FGD). Our observations reveal a unique pattern of interconnectedness between SDGs that can be related to COVID-19 consequences. Qualitative interpretations of focus group discussions also depict, that additional spillover effects can be obstacles for achieving SDG 5 (Gender Equality), SDG 9 (Infrastructure & Innovation) and SDG 10 (Reducing Inequalities), SDG 17 (partnerships for the goals), SDG 11 (sustainable cities). Therefore, we consider the interdependent implications and recent trends in international development related to sustainability as a useful framework in the post-pandemic recovery period.

## Introduction

The world has committed to implementing the 2030 Agenda for Sustainable Development with 17 Sustainable Development Goals (SDGs) adopted by United Nations (UN) Member States in September 2015 [1]. The unusual situation created by COVID-19, in early 2020, is influencing this commitment and undermining the general approach toward suitability by slowing down the process toward achieving the 17 SDGs and changing the trajectory of development. The overarching aim “leave no one behind” is threatened by the current growing inequalities. While the multiplied global challenges, economic and financial shocks associated with COVID-19 make financing for sustainability even more difficult [2].

The impact of the pandemic on society is unclear, long-lasting, and difficult to measure. Consequently, contributions to the literature and research in this aspect are a necessity. This study analyses critical aspects regarding the global pandemic’s impact on the achievement of the SDGs and highlights the interdependencies between the goals in light of the new circumstances through a synopsis of the current literature, empirical observation and analytical evaluation based on problem-oriented qualitative group discussions of selected scholars and practitioners. This study relies on the SDG literature and considers the wide context framed by the pandemic crisis to explore interdependencies among the most immediately affected SDGs.

The article brings a contribution in several ways. First, it puts forward an analysis of the impact of the COVID-19 pandemic on the SDGs. Second, the research brings together and relies on the insights of scholars and practitioners from different fields and different levels of analysis. Third, the insights generated contribute to furthering our understanding of post-COVID-19 recovery.

The study is structured as follows. First, an overview of the global evolution of the SDGs, their forthcoming nature in the COVID-19 pandemic and post-pandemic recovery is provided. Second, observations and reflections related to the pandemic’s consequences for the sectors of society that were the most immediately affected by the pandemic [3], such as health (SDG3), education (SDG4), economy (SDG8), consumption/production (SDG12) and climate (SDG13), are presented. Thereafter, interconnections between SDGs are analyzed to identify synergies that can guide the integration of the 2030 Agenda for Sustainable Development in recovery processes. Finally, recommendations are presented as a way forward.

## Synopsis of the current literature on the impact of the COVID-19 pandemic on SDGs

The SDGs have transformed global efforts to foster development and enhance sustainability. The SDGs represent, in many ways, a departure from previous approaches to doing development, emphasizing the universality of assumed targets, a much wider thematic agenda, new central actors, and an important focus on financing for development. All of these are deeply affected by the COVID-19 crisis. The new 2030 Agenda has become more attractive and inspiring than many commentators would have initially assessed. In certain ways, the global endorsement for the SDGs [4] is both unexpected and hard to explain, given past reactions to proposals of intergovernmental organizations, including the previous Millennium Development Goals (MDGs). Few aspects can partially explain such enthusiasm.

First, the creation of the SDGs was a co-creative process that allowed many voices to participate, enabling a widespread feeling of ownership. Second, the SDGs are claimed to be universal [5]; that is, they are applicable to both developed and developing countries, which brings a long-awaited perception of equality between wealthier and developing countries. Third, there is a wider commitment to certain global challenges (e.g., climate change, poverty, water, peace). The encompassing nature of the SDGs enables them to address the values of multiple groups and, in consequence, gather support for the 2030 Agenda.

The universality of the SDGs does not come, however, without challenges. While involving everyone both in generating the goals and in implementing them is inclusive, it also comes with limitations around flexibility, much needed for adapting the SDGs to local contexts. This aspect relates to the broader discussions on post-colonialism and global impositions that make a key point. For development to be owned and not perceived as an imposition, it needs to be generated locally, in local terms, and based on local vision. Despite such shortcomings, the SDGs have the potential to provide our communities with an encompassing framework that can guide our thinking and imagination to envisage potential better futures. COVID-19 arrested for a moment the advancement of Agenda 2030 and affected the achievements for SDGs.

### COVID-19 and the SDGs

Global phenomena that represent a core focus of the SDGs have been significantly altered, drawing our attention to new realities and ways of life we did not imagine before. Mobility and migration are heavily affected through lockdown measures with significant human and economic costs [6]. The pandemic triggers an economic crisis of large proportions with augmented impact on developing countries [7], putting a large number of people in poverty, for the first time in three decades poverty is increasing [8]. The economic consequences are significant and widespread, affecting all areas of the economy, including capital flows, business operations, employment and jobs [9]. Education is forcefully digitalized [10], impacting over 1.2 billion learners in over 170 countries (72% of all learners) during the initial lockdown [11].

Last but not least, the decreasing trend in democratic government conduct is accentuated as states of emergency were imposed during the first wave and are recently reinstated as the second wave of the pandemic is emerging [12]. Many of the above global trends pre-date COVID-19, being accentuated as the pandemic progresses, including digitalization, authoritarian and extreme right tendencies, and growing global inequality [9]. We were already behind on reaching the SDGs before the COVID-19 pandemic started [13]. In essence, the pandemic is bringing tension at a global level by giving on the one hand reasons for closing borders, restricting the movement of people and goods, and authoritarian actions, while on the other hand requiring global collaboration for tackling the pandemic, which is inherently global.

In terms of positive consequences, the multilateral system is enhanced by the pressures for collaboration brought by the pandemic. Additionally, advancements in digitalization and consolidation of health, educational and social services will bring long-term benefits. From an environmental perspective, limited mobility brings improvements in air quality and decreases CO_2_ emissions, having positive consequences, at least in the short term [14]. Although somewhat contradictory, the SDGs in the COVID-19 context guide and encourage mitigating the negative impact of the pandemic and provide a framework where much-needed leadership and responsibility can unfold.

The timeframe for SDGs implementation entered the “Decade of Action” until 2030, but the SDGs are far from being on track. In light of the pandemic, the “Decade of action” should be re-launched as a ‘Decade of Recovery and Action’ using the SDGs as a global framework [15]. Due to the unexpected crisis, the shift of Member States’ priorities, which are also the main entities responsible for the implementation of the 2030 Agenda, is diminishing the efforts to reach the SDGs. However, to avoid risking the progress achieved to date [16], the crisis can be used as an opportunity to strengthen commitment to the 2030 Agenda. By placing sustainable development at the core of recovery plans, a better response to future crises is enabled, implying stronger health systems, fewer people living in extreme poverty, less gender inequality, a healthier natural environment, and more resilient societies [2]. Balanced ecosystems are important for disease control [17], which highlights the significance of understanding the interdependencies between people and ecosystems [18].

### Interconnections between the SDGs

The International Monetary Fund (IMF) has predicted that the global economy will decline by 3% in 2020, with advanced economies declining by double that amount [19]. This threatens the achievement of SDG 1 because despite some countries making progress in decreasing pre-pandemic income inequality, a global recession could cause approximately 71 million additional people to live in poverty [20]. The achievement of SDG 10 (reduced inequalities) is negatively impacted because the most vulnerable groups (women, youth, low-wage workers, small and medium enterprises, informal sector) [3] have to cope with the most damaging impacts of COVID-19 and the current growing inequalities between countries.

Problems created by the pandemic emphasize the indivisible character of the 2030 Agenda for Sustainable Development. Multidisciplinary systems-thinking is needed for exploring interconnections between environment, wildlife, and humans all in one health approach [21]. The combination of public and business policies with technological innovation can mitigate the negative impact of economic activities on the SDGs [22]. The dynamism of urban areas can also be sustained by proper conservation policies and deployment of ecosystem services [23]. Furthermore, it is essential that plans for achieving resilience be able to cope with global stressors and disruptions such as pandemics [24].

It is widely accepted that the 2030 Agenda for Sustainable Development is indivisible and that the implementation of one Goal has positive or negative effects on the others [25]. Scholars have analyzed SDG interactions using diverse methods, i.e., Goal Interaction Scoring on a Seven-Point Scale [26, 27]; The Sustainable Development Goals as a Network of Targets [28]; Cross-impact matrix at the Target’s level [29]; Integrated Assessment Models [30]; Empirical work using network analysis for assessing SDG interactions [31], etc. To date, the analyses of interactions between SDGs mainly rely on expert opinions [32]. Previous research by authors has analyzed the interconnection between the goals and targets in the context of multi-stakeholder networks [33], identifying multidimensional aspects of target 4.7 (Education for Sustainable Development) to be strongly connected with SDG13, SDG7, SDG11.

### Policy response and green recovery

It is estimated that global investment in recovering from COVID-19 will equate to $20 trillion. The choices surrounding how this money is invested will affect generations for decades to come and determine whether communities become more resilient to future disruptions [34]. Green refers to rebuilding after the COVID-19 crisis in a way that tackles climate change and aligns with the SDGs. This requires an interconnected and comprehensive approach to policy implementation and recovery planning where countries can succeed in transitioning to green economies and build resilience to future disruptions [35]. Green recovery will be carried out differently by country because measures depend on factors such as macroeconomic conditions, fiscal budget, pre-existing stimulus packages, capacity and ambition to address the climate crisis, and level of commitment to other policy objectives [19]. These factors demonstrate that action needs to be matched by capacity, and therefore, capacity building is integral to green recovery [36]. Green economies should be inclusive and equitable, generate poverty reduction and growth, create new jobs and encourage stakeholders to act environmentally responsibly [36]. Although each country’s plan will vary, there are common principles to be considered.

*Green stimulus and the creation of jobs*

Investment choices are integral for moving towards more sustainable systems and practices [24]. There has been increasing demand for “green stimulus” that will restart the economy while simultaneously facilitating a transition to more sustainable systems. The European Commission’s growth strategy has prioritized green fiscal stimulus which due to COVID-19, has required increased funding, with 25% being allotted towards green expenditures. The IMF and International Energy Agency have announced similar plans for green expenditure.

This level of investment in the green economy is comparable to the American Recovery and Reinvestment Act (ARRA) of 2009 designed to harness economic benefits from investing in green infrastructure, promoting renewable energy and creating green jobs after the recession. Important lessons can be learned from this Act, as the areas that benefited the most in the US from green stimulus were the areas that had the highest proportions of green skills. In areas that had less than the median number of occupations requiring green skills, green stimulus was ineffective. The ARRA also showed that green stimulus benefited those employed in high demand “brown” occupations such as the oil and gas industry because these jobs share some of the skills needed for green jobs. Training was still required but was more likely to be successful. For workers in low-demand brown jobs, green stimulus investments were less beneficial because these jobs do not share many green job skills.

This demonstrates the importance of making investment decisions based on the skills within each community and, although costly, the need to combine investment in green job creation with investment in training. Green stimulus should be seen as a long-term response to the pandemic because it is a tool for reshaping the economy rather than restarting it. When applied in isolation, it cannot provide quick assistance to those employed in roles that do not require green skills, and these roles were the most negatively impacted by lockdowns during the pandemic. However, as a long-term response, it is necessary because investments in infrastructure and transport should reflect behavioral changes, such as more people working from home. It is recommended that research be carried out to gain data on which workers would benefit from green skills training and whether training is a viable option to help workers transition from jobs in the brown economy to jobs in the green economy [19].

To date, analysis by the Organisation for Economic Co-operation and Development (OECD) on green recovery responses shows that governments have included the measure of “creating jobs and stimulating economic activity through ecosystem restoration” however skills training has not been prioritized. The OECD advises that investment in skills and education is vital to ensure a successful transition to a green economy that is inclusive of all communities. In this way, the UN’s aim of “leaving no one behind” should be upheld by ensuring that all individuals have the opportunity to gain the skills needed to transfer to greener sectors and therefore benefit from a green economy.

Shortages of green skills are most prevalent in sectors including renewable energy, resource efficiency, building renovation, construction, environmental services and manufacturing. This issue is most problematic in developing countries. It is important that strong partnerships between the government and industry be developed to make funding decisions, which will reduce skill gaps and meet the demands of the green sector [37]. The New Climate Economy report cites transitioning to more sustainable systems as a way of increasing employment in low carbon sectors by 65 million people by 2030. When considering the jobs lost during the transition, the net gain is 37 million jobs. The International Labour Organization (ILO) has estimated that capping global temperature increases at 2 °C would create approximately 24 million jobs by 2030. However, the OECD warns that research on the relationship between employment and green policies is scarce because transitions to a green economy are lacking in number, which affects the ability of econometric analysis to accurately predict changes in employment figures [19]. The UN has highlighted the importance of using reliable data and statistics to make informed decisions about responding to COVID-19 while achieving the SDGs [2].

*Investment in green innovation*

It is important that the cost of transitioning to a green economy is affordable to avoid future generations being burdened with debt. Currently, one of the major barriers to transitioning to greener systems is the underpricing of fossil fuels by G20 economies. Public spending should reflect a long-term commitment to green innovation in the private sector. This should be accompanied by pricing reforms. Taxing the damage caused by carbon emissions and environmental pollution and eradicating fossil-fuel subsidies could create government revenue that could then be diverted to investments in the green economy. In 2015, government subsidies from 16 major economies to the fossil fuel industry amounted to $3.6 trillion, which was 8.6% of GDP on average. Reliance on fossil fuels inhibits the achievement of many of the SDGs, such as Goals 7, 11 and 15 [19].

Incentives for long-term investment in renewable energy decrease the demand for fossil fuels and aid the transition to more sustainable economic practices. “Technology spillovers” can act as a catalyst for innovation. A technology spillover occurs when the research and development of a firm or industry rapidly diffuses to other firms and industries. This effect has the potential to transform an industry via knowledge exchange, learning and the development of skills. However, spillovers can act as a disincentive to private firms and industries because the firm or industry carrying out the research and development receives little or no return from the diffusion of innovation despite incurring costs.

Technology-push policies are a solution for addressing underinvestment in green research and development. An example of this involves government subsidies designed to increase demand for clean energy. Subsidies would trigger more investment in the research and development of technology and scaled up production, which would cause clean energy to be cheaper and more widely available. It is advised that subsidies are phased out once demand has increased, as an initial increase in demand will cause demand to snowball.

Diverting subsidies from fossil fuel investment can also result in more revenue available to invest in green services such as transport systems. However, it is essential that additional costs caused by increasing tax and lowering subsidies in the fossil fuel industry are not passed on to consumers. If consumers were to bear the cost, this could increase energy poverty and inequality and damage the effort to achieve the SDGs. Accessible clean energy is vital for a higher quality of living [37].

A strategy for increasing government revenue for investment is lowering tax avoidance. Low-income countries lose 1.3% of their GDP via companies practicing tax avoidance each year, which highlights an opportunity to intercept this money and use it to fund the achievement of the SDGs. However, it must be noted that this increase in revenue might be offset by companies incurring loss of income and bankruptcy due to the pandemic [24].

*Behavior and opinion change*

Green recovery policies should consider how the pandemic has caused changes in behavior and opinion. This includes adaptive behaviors such as using unfamiliar technology to work remotely and reduced use of public transport and private vehicles. It is estimated by Workplace Analytics that one-third of the global workforce will maintain working from home practices either part time or permanently. In addition, it is predicted that air travel will return to pre-pandemic levels at a slow rate because it is thought that business travel will never return to the same level.

Post-crisis investment should consider high-speed broadband and teleworking infrastructure as well as domestic energy efficiency for those working from home on a long-term basis [38]. An increase in the number of people working from home has led to a decrease in greenhouse gas emissions, so governments must use this momentum in green recovery [24]. Sustainable energy transitions depend on complex behavior changes, such as working from home or cycling, so there is certainly an opportunity for successful de-carbonization of energy systems [39].

Opinion change refers to the likelihood that the public will be more supportive of green policies due to COVID-19. This is because the pandemic has powerfully demonstrated that issues that appear remote can quickly escalate and have wide-reaching effects. Second, preparedness is vital and requires years of work to be successful. Third, the cost of not being prepared outweighs the cost of preparation [40]. A study by the UN asked one million people about their aspirations for the future, and people around the world are citing climate action as being important to them in future COVID-19 recovery plans [40].

*The circular economy and nature based solutions*

OECD analysis shows that 24 national governments plan to implement pandemic measures that have negative environmental impacts. This includes allowing single-use plastics, lowering standards on water and air quality, reducing taxes related to the environment, granting bailouts and subsidies to fossil fuel-intensive industries such as aviation and increasing subsidies for fossil fuel-intensive infrastructure. Some governments have also implemented temporary consumer measures such as reducing or subsidizing electricity bills. Currently, the non-green measures being implemented outweigh spending on green measures. Rhodium Group found that EU spending on green measures equates to 20% of total spending, but this percentage is lower elsewhere. In addition, the “Green Stimulus Index” has highlighted that 14 of the 17 countries studied assign government revenue to environmentally damaging industries, which will outweigh the impact of green stimulus [37].

The Ellen MacArthur Foundation has calculated that transitioning to a circular economy has the potential to create 700,000 jobs by 2040, reduce greenhouse gas emissions by 25% and save US$200 billion per year. A circular economy would be more resilient to global disruptions such as pandemics and aid the achievement of the SDGs because it addresses pollution and climate change while creating jobs [41]. UN High-level Political Forum (HLPF) in 2020 identified nature-based solutions and circular economy as important for achieving the SDGs and argued they should be mainstreamed by decision makers to create more resilient systems.

The Ellen MacArthur Foundation has also cited the circular economy as the key to creating resilient supply chains post COVID-19. The majority of supply chains are transnational; sourcing raw materials, manufacturing and then delivering the end product to the consumer tends to happen across multiple countries. Including the reuse and recycling of materials in supply chains can lead to cheaper products for consumers and cheaper materials for producers. In the context of the pandemic, strict hygiene rules would need to be adhered to, but it is certainly an opportunity to redesign supply chains for a more resilient economy [41].

The World Economic Forum has launched a set of 21 metrics that are designed to show how companies are tackling the climate crisis and contributing to the SDGs. The metrics are split into four themes: principles of governance, planet, people and prosperity. The aim is that investors will be more attracted to companies that are performing well according to the metrics. This increases transparency in the private sector and encourages companies to be more sustainable [42]. Nature-based solutions provide a strategy for protecting ecosystems while simultaneously overcoming societal challenges and positively impacting wellbeing and biodiversity. The SDGs encourage and complement the implementation of nature-based solutions [43].

*Capturing co-benefits*

Capturing co-benefits is an important aspect of green recovery [38]. Governments must fund short-term emergency packages as well as long-term green recovery policies. Investing in green systems and clean energy will be challenging, as it is estimated that before COVID-19, governments were $2.5 trillion short of being able to achieve the SDGs. Therefore, it is recommended that experts in decision science be consulted to perform cost–benefit analysis to aid decision makers in which SDGs should be prioritized. The SDGs facilitate this approach as many overlap and the achievement of one has a positive effect on the achievement of another. Each SDG and target should be assessed in terms of the level of priority post COVID-19, the extent to which it facilitates development rather than just growth and its ability to create resilience to global shocks and stresses [24]. Policy makers must focus on maximizing the positive impacts of each decision made by assessing potential positive externalities and the needs of each community. For example, in lower- and middle-income countries, investing in renewable energy also increases the rates of electrification, which lowers poverty levels and makes neighborhoods safer [38].

## Research design and analytical framework

This section will present the way methodology-related decisions were made and how the analytical framework is structured. The aim of the study was to better understand in how far SDGs and their interconnectedness are specifically affected by COVID-19 pandemic and the related crisis management. Since this research interest was following an ongoing global phenomenon where valid quantitative data were not available for cross-sectional analysis, we applied a qualitative research approach. We set up structured and moderated focus group discussions (FGDs) [44] to gain data for problem-oriented qualitative analysis. FGDs were facilitated through demonstrative expert speeches [45] to provide an impulse for the discussions, which authors call “Impulse Speeches”. We secured procedural reliability and communicative validation throughout the analytical process using the following measures and instruments:

- The study is based on the result of the project “Effects of COVID-19 on the Sustainable Development Goals”, which was funded by iac Berlin. Involved scholars and practitioners were chosen as experts following two criteria: (a) experts had to be members of the SDG group of the Bosch Alumni Network, (b) expertise should respond to the sectors most immediately affected by the pandemic. The experts belong to different sectors (policy, business, academia, nonprofit, etc.) and different countries (Italy, Mexico, North Macedonia, Romania Albania and Germany). Detailed information about experts is displayed in Annex.
- To cover the most relevant SDGs identified through the initiating literature synopsis, the experts organized seven online sessions during July–October 2020, consisting of FGDs that followed a standardized procedure. More specifically, we conducted dual moderator FGDs following the procedure suggested by Krueger and Casey [46] with a division of moderating roles (facilitator using impulse speeches to initiate thematic discourse and moderator formally conducting the FGD). Experts chose the focus of each session concentrating on one or several SDGs. Following a speaker’s impulse of 25 min, researchers conducted a moderated and problem-oriented FGD that was guided by a standardized catalogue of questions and equal time for discussions. Each impulse speech condensed specific research findings, observations and case-based examples related to selected expertise. A video documentation of each session was distributed to the experts to provide the database for a follow-up qualitative analysis based on critical individual content reflection. This was again backed up by experts’ discourses in one finalizing session for communicative validation of interpretations. The final session was set up to systemize the findings on interconnectedness.
- Analyses follow the logic of the DPSIR framework (Driving forces; Pressure; State; Impact; Response) (developed in 1999 by the European Environmental Agency for integrated environmental reporting and assessment). The simplified DSR framework, previously implemented by the OECD for environmental indicators for agriculture where the ‘‘pressure’’ component is replaced with the concept of ‘‘driving forces’’ [47], is used for the purpose of this study.
  1. Driving force (D), (pandemic crisis context, alike for all involved countries)
  2. State (S), (new dependencies created or enforced between SDGs. For each of the selected SDGs, the most evident key implications on other SDGs, as triggered by the pandemic are identified)
  3. Response (R), (policy response considering the SDG dependencies and study findings)

The interactions between Sustainable Development Goals and Targets can be more visible through real case observations [48]. According to “Deciphering the scientific literature on SDG interactions: a review and reading guide” [49], there are seven types of SDGs interactions identified in several studies as follows: (1) goal–goal interactions; (2) target-target interactions; (3) indicator-indicator interactions; (4) policy-policy interactions; (5) goal/target/indicator and/or policy interactions; (6) external entities and (7) geographic location. This study considers goal–goal interaction. Table 1 below displays the research design, approach and methods of the study.

**Table 1:** Study approach and methods

DSR framework Driving force (D: COVID-19 pandemic)	Sections	Subsections	Approach	Methods
State (S)	4) Results: SDGs interconnection analyses	4.1. The Impact of COVID-19 on the SDGs and their interconnections	Conducting 2 online FGDs about general impact of the pandemic for the SDGs Conducting 4 online FGDs with facilitating impulse speeches by selected experts, reflecting the impact of COVID-19 on the SDG4; SDG12; SDG8 and SDG3; and SDG 13	Qualitative analyses of group FGDs based on authors impulse speeches, observations and reflections
State (S)		4.2. Interconnections of SDGs triggered by pandemic	Conducting final online FGD to discuss the results of individual sessions and to reevaluate how far the analyses represent the group discussions For each of the 5 SDGs (SDG4, SDG12, SDG13, SDG8 and SDG3) the interconnection with the other remaining 16 SDGs is analyzed, in order to identify the most interconnected SDGs, fostered by pandemic for policy recommendation	Qualitative analysis of all group-discussions; concluding communicative validation; systematization of results
Response (R)		4.3 Green recovery: the way forward	Conducting final online FGD to discuss results. Some aspects from the findings were elaborated in order to channel policy response and a way forward	Based on systematization of results of qualitative analysis

## Results of SDGs interconnections analyses

This section contains the study results and discussions, divided into three parts. Section 4.1 presents the results of the author’s impulse speeches. Section 4.2 presents the analysed interconnections between SDGs as the result of problem-oriented FGDs, and Sect. 4.3 argues for a potential policy response in the form of green recovery.

### The impact of COVID-19 on the SDGs and their interconnections

This subsection will present the results of the four impulse speeches regarding the impact of the COVID-19 pandemic starting from SDG 4, SDG 8, SDG 3, SDG 12 and SDG 13. The results depart from everyday practices and experiences during the pandemic. Analyses build on the observed practices and form the basis for our understanding of how SDGs interconnect and are affected by the pandemic.

- Rethinking environmental education in the pandemic
  Many educational organizations rely on international projects for traveling and exchange, among other activities. Due to lockdown and travel restrictions, many projects have been delayed or canceled. This made stakeholders rethink if traditional project procedures had to be continued under conditions of COVID-19. Questions were raised as follows: Is it truly important for young people to travel around for short-term projects? How can one better correlate international work with local communities? What are some of the advantages and disadvantages of educational online programs?
  Organizations were pressured to find solutions, such as “QG Enviro”, a local organization in Lecce, Italy, working on environmental education and awareness, which is reorienting toward sustainable agriculture and tourism for resilience to unexpected events. The organization was created in 2017 by three young professionals in the natural sciences who were willing to help local youth improve knowledge and skills related to the environmental and agricultural fields and raise the quality of environmental education in the area.
  The main activities carried out by the group are youth and best practices exchanged through Erasmus + projects, international projects related to sustainability and ecosystem restoration, and local activities with children and adults. During the first 2 years of camps, courses and trekking were organized mainly targeting adults and kids coming from the Apulia region.
  In 2019, QG Enviro started its participation in several different EU and international projects, with some of them still ongoing. The founders embraced the idea of giving locals some more opportunities in widening their potential and knowledge through exchanges with other organizations coming from abroad. At the end of 2019, QG Enviro was ready to take more actions and widen its impact on local communities in Apulia.
  With the COVID-19 pandemic, everything changed. Their two main EU projects were delayed, many others did not start at all, and all the local activities were shut down. The organization was able to migrate much of the work online. Strong bonds were created in 2020 because of the situation, with founding organizations being open to innovation. As such, two projects were transferred completely online with major changes. The time spent in full lockdown, despite the personal difficulties for all members, was spent well in rethinking activities. The group had the unique chance to stop and look deeply at its mission as a team, finding and untying many knots in internal processes. Alessia Di Seclì, president and founder, describe the current situation:
  “The lockdown at the end was an “incubator” for our metamorphosis as an organization. Just imagine that now, we are preparing to run 2 online courses on regenerative thinking, we are working on a local community’s development plan and we are going to cooperate with other organizations in the migration sector, giving them our knowledge to create professional courses for young migrants coming in Italy. We are trying to see this as a pivotal moment to change, adapt and even grow in a different manner. What makes us so optimistic is our high flexibility and the help of the people we are surrounded. We know many others are in critical conditions, let us think to people working in tourism, especially here in Italy. But at the end we need to accept that we cannot go back to normal and we must embrace this new time that is coming.”
- Forced home-office work and its effects on health, gender and inequality
  This section addresses the work-related impacts of COVID-19 pandemic management—mainly referring to forced home-office arrangements in Germany. Before the beginning of the pandemic, approximately 40% of employed workers in Germany had the opportunity to use home-offices as a self-chosen work flexibilization option, whereas approximately 60% had no home office option. During the 2nd quarter of 2020, this relation was inversed—with the lockdown regulations suddenly turning workers’ flexibilization option into a home-office obligation [50].
  Handling the lockdown most efficiently while obtaining productivity demanded technological adjustments on different levels, such as telecommunication infrastructure, cloud technology, and workplace technology. In addition, encompassing adjustments in labor relations and laws needed to be undertaken in a rather short timeline. Organizations needed to quickly establish a new digital working culture as well as appropriate virtual leadership. Finally, the individual worker in a forced home-office environment had to ensure that local working conditions, personal computer skills and digital competences were set up to meet the new requirements of digital cooperation and co-creation. Fadinger and Schymik [51] have recently delivered evidence that sending workers to the home-office during the COVID-19 pandemic helped decrease infection risk in Germany. This is a positive result of labor-related crisis management with respect to securing the “Good Health and Well-being” (SDG 3) of employees and their social environment.
  However, at the same time, workers found their professional, their social and their private lives more than ever forced into a digital (working) environment, while the radicalness of the change towards home-office-work did not allow enough time to cope with challenges appropriately. This large-scale invasion of work into the private sphere accompanied by increased technological complexity created new stressors for the individual dealing with the situation—in addition to coping with the radicalness of the imposed change as such. While some workers found themselves and their home-office conditions set up adequately to achieve their tasks, many others were confronted with various obstacles, including the missing of separable home-office working space, technical difficulties, work overload, extra-burden through child-caring, home-schooling, or caring for dependents [52].
  Given these conditions, members of organizations are often willing to invest individual extra efforts to achieve the common goal of surviving the situation [53]. The downside of such an extra effort combined with the negative effects of increased technology use can be a decrease in well-being, if coping resources are few, a goal line and improvements are not in sight, and rewards are pending [54]. When exploring the effects of the COVID-19 pandemic on the SDGs from a broader scientific perspective, such potentially negative effects of forced home-office need to be taken into consideration. The current YouGov omnibus reports that approximately 1/3 of the panel named negative aspects related to working from home, such as work invasion into the private environment (31%), technological complexity and poor user experience (29%), difficulties communicating and cooperating with colleagues (28%), or poorer concentration (20%) [55]. Other current studies are raising concerns about gender inequalities (SDG5) in experiencing the COVID-19 lockdown in Germany [56, 57]. Bradbury and Isham [58] note the consequences of COVID-19 on domestic violence.
  In March 2020, one of the authors launched a scientific study, monitoring, if and in how far forced home-office during lockdown and thereafter induces technostress [59–61], ultimately leading to cognitive and emotional irritation [62] and decreased user well-being. In addition, the study addresses whether participants are experiencing unusual burden (i.e., home-office in combination with child day care or home schooling). To determine to what extent individual resources could positively moderate the technostress-irritation relation, digital competence and self-efficacy were also measured. The study is still ongoing in February 2021 and covers the “lockdown light” period that started November 2nd 2020 and the “hard lockdown” starting from December 2nd 2020, including almost 1000 German workers who had been forced into home-office work.
  The first data analyses reveal that the higher the level of technostress, the higher is the level of psychological strain (cognitive and emotional irritation) in general. A striking finding is, however, that the increase is higher for those who experience extra burdens during lockdown and forced home-office. Other results are alarming as well. Under conditions of high techno-stress related to forced home-office, one of the strongest predictors of happiness and mastery experience in life (self-efficacy), which is again related to good and long lasting health, does not have a significant positive moderating effect on the technostress-irritation relation. A similar finding accounts for digital competence as another important individual stress-coping resource. Preliminary interpretations allow a first conclusion that technostress induced by forced home-office work can be considered a substantial threat to decent work and to workers’ good health and well-being.
  The survey also shows that the work-related effects of pandemic crisis management might have enhanced socio-structural injustice and inequality. This allows us to draw a conclusion on interconnected effects regarding SDG 3 (Good Health and Well-Being), SDG 5 (Gender Equality) and SDG 10 (Reduced Inequalities). Finally, the radicalness of forcing workers into home-office as a measure of the COVID-19 pandemic crisis management in Germany was simply too much to handle for many employees—even for individuals with higher personal resources such as self-efficacy and digital competence. Putting these circumstances together, the measure of forced home-office under the umbrella of COVID-19 pandemic management deserves special and continuous interest to further investigate whether it might interconnectedly affect SDG 3 (Good Health and Well-Being), SDG 8 (Decent Work and Economic Growth), SDG 5 (Gender Equality) and SDG 10 (Reduced Inequalities).
- Implications for consumption/production
  SDG 12 “Ensure sustainable consumption and production patterns” is one of the most transversals in its design because it includes a wide range of topics, such as efficient use of natural resources, minimizing the loss and waste of food, ecological management of chemicals throughout their life cycle, solid waste management, sustainable public purchases, sustainable tourism, environmental education and finally the elimination of fiscal incentives for fossil fuels, which can distort the market. It allows a broad spectrum of actions and the participation of actors from multiple sectors.
  Goal 12 is highly related to both the producers and consumers, indicating the dependencies of better products and the increased awareness of the demanders. The pandemic appears to have cast a spotlight on systemic inequity; for instance, Latin America and the Caribbean are in a weaker position than many other countries.
  Before the pandemic, ECLAC (Economic Commission for Latin America and the Caribbean) predicted “that the region would grow by a maximum of 1.3% in 2020.” Due to the effects of the crisis, a fall in the GDP of at least 1.8% is expected [63]. Moreover, as a consequence of self-isolation and social distancing, decreased working hours and wages, the demand for goods and services is reduced. The impact in many economic sectors, mostly in services that are highly interrelated with gatherings, is significant.
  At the same time, it can be an opportunity to shift the way things are done. The consumer market is more fragile because of the pandemic [64]. People are more interested in their impact, buying more locally to help companies and individuals nearer to their communities. The sensitivity of the consumers is increased, having in the magnifying glass companies and public sector, demanding more responsibility for their actions and recovery plans, which is either applauded or taunted.
  It does not mean that consumers now have a complete understanding of sustainability, but it puts the conversation back to the table like never before. People are now also worried about the social impact, not only about the environment. Employers asked their employees in Mexico for a month of vacations instead of supporting them economically during the lock down, which elevated the conversation on social media and had a negative impact on the community (ejecentral.com.mx). Finally, even sales fell drastically, and some shops had to be closed.
  The pandemic conditions are forcing businesses to innovate and re-evaluate the way they operate. For example, companies such as GM and Ford switched out from pickups to breathing machines [65]. For traditional companies, it will be difficult to survive. Although the pandemic has accelerated some innovative changes and a trend toward sustainability, sustainable business models are still not mainstreamed.
- Global implications for mobility and climate
  The pandemic has accelerated environmentally friendly mobility measures, and a shift in public mobility and “car-free cities” is being highly discussed in policy circles. During the lock-down air pollution dropped by 60% globally [66], and bike use increased by 129% [67]. Communities have the tendency to become more resilient and independent in the face of unexpected conditions. Cycling makes communities more resilient [68], and taking liability and equality as a starting point, infrastructure development with short-term responses creates long-term change. The “*Shift-Avoid-Improve*” principle [69] emphasizes the creation of a ‘*car-free*’ generation by 2025.
  Individual decisions are important for the mobility system, in order for the trend to continue and SDG 11 about the sustainable cities and communities to be achieved. The mobility behavior of citizens is changing. According to McKinsey’s and Company [70] global consumer survey, 52% of respondents travel less, the use of private micro-mobility is increased by 9% and that of shared micromobility is increased by 12% compared to pre-pandemic levels. Alternative transport, such as cycling, allows easy social distancing and contributes to better public health through the reduction of air pollution. However, many residents perceive public transport as posing the risk of being infected and are using more private vehicles. Despite changes in travelling time, reducing the risk of infections is the main reason for travelers’ choices [71].
  Cities around the world are turning to non-motorized transport alternatives to help solve congestion and pollution issues [72]. For example, Brussels, Milan, Montreal, New York, San Francisco and Seattle have each introduced more than 20 miles of dedicated cycle paths, and Paris is in the process of investing $325 million to update its bicycle network [70]. This paradigm shift demands new infrastructure that serves and boosts local cycling. In anticipating future needs, big data can lead to better decisions and improve outcomes and evidence-based policy making. It is expected that between 2020 and 2025, autonomous driving, connectivity, electrification, shared mobility, non-motorized transport policies [73] and regulation of private car-based mobility will be on the rise.
  Before the pandemic, the majority of countries had not been on track to achieve lowering greenhouse gas emissions targets [74]. The global response to the pandemic has caused a sharp decrease in greenhouse gas emissions and an increase in air quality. CO_2_ emissions are predicted to decrease by 8% in 2020, which would bring CO_2_ levels to those of 10 years ago. However, it is expected that the long-term impact will be non-existent if atmospheric CO_2_ levels continue to increase after the pandemic [37]. In addition, due to global SO_2_ emissions decreasing by 20%, the impact of aerosol cooling is lowered, and therefore, the positive environmental benefits caused by the pandemic will be minor [24].
  The global response to COVID-19 could accelerate progress on climate change if governments are able to use the momentum created by the short-term decrease in global greenhouse gas emissions. Governments must avoid an increase in emissions once restrictions on movement are eased [38]. If G20 countries were to simply recover the existing “brown” economy, this would lead to permanent environmental damage [19], as a “business as usual” approach could cause a 3 °C increase in global temperatures by the end of the century [38]. Therefore, there has been a shift from focusing on the immediate crisis to “building back better” by ensuring a “green” fiscal approach and investing in a transition to greener systems.
  Opinion polls in a number of countries show that mandatory and self-imposed restrictions on work and travel have led to people noticing cleaner air, less traffic on roads, a reduction in noise pollution and the return of wildlife. This has triggered people to question whether returning to “normal” is an adequate desideration after COVID-19. This marks an opportunity for transitioning towards green systems [38]. A study by the WHO has concluded that 80% of those who live in urban areas are exposed to levels of pollution that are harmful [75]. Studies have shown that an increase in particulate matter of PM2.5 can increase the death rate of COVID-19 by 8–16%. In addition, socially disadvantaged groups tend to be more exposed to air pollution, which means they are more susceptible to the cardiovascular and respiratory complications associated with COVID-19 [37].
  The above section presented four case studies, reflecting on how COVID-19 impacts the SDGs. The next section will focus more specifically on the interconnections between the SDGs.

### Interconnections of SDGs triggered by the pandemic

This section comprises the results of the analyses of the individual sessions. Analyses of this study consider the pandemic as a driving force to identify which SDGs have stronger connections in this context by analyzing links of each of the 5 SDGs (SDG 4, SDG 12, SDG 13, SDG 8 and SDG 3) with the other remaining 16 SDGs. (For example, SDG 4 has stronger links with SDG 10, SDG 9, SDG 13 and SDG 5). Detailed information for each of the connections is displayed in Table 2.

**Table 2:** Key implications for the SDGs under research (SDG 4; SDG 12; SDG 13; SDG 8 and SDG 3) for other SDGs, as highlighted by the COVID-19 pandemic

SDGs under research	Key implications to other SDGs	Explanation
“Quality Education” (SDG 4)	SDG 10 “Reduced inequalities”; SDG 9 “Industry, Innovation and Infrastructure”; SDG 13 “Climate Action”; SDG 5 “Gender equality”	Switch to Online learning, highlighted the unequal access to knowledge and educational resources, even between genders; Increased demand for access to technological infrastructure, technological resources and innovation; travelling restriction, related to educational activities, and physical exchanges reduced mobility
Decent Work and economic growth (SDG 8)	SDG 3 “Health and Well-being”; SDG 4 “Quality Education”; SDG 5 “Gender Equality”; SDG 10 “Reduced inequalities”	Smart working related health problems (posture defects/overweight/diabetes/short sightedness/sleep disturbance/addiction/risk behaviour); insufficient self-management/poor digital competences; forced homework can increase social-cultural injustice; job loss, reduced working hours
Responsible consumption and production (SDG 12)	SDG 1 “No poverty”; SDG 9 “Industry, Innovation and Infrastructure”; SDG 15 “Life on Land”; SDG 4 “Quality Education”	Job loss/reduced working hours; pressure to innovate for businesses; less use of resources; education for sustainable development
Climate (SDG 13)	SDG 11 “Sustainable cities”; SDG 3 “Health and Well-being”; SDG 8 Decent Work and economic growth; SDG 15 “Life on Land”	Private cars/cycling; reduce business travelling/air quality; home working/reduce of physical contacts; responsible use of resources
Health and Well-Being (SDG 3)	SDG 1: No poverty; SDG 8 Decent Work and economic growth; SDG 10 “Reduced inequalities” SDG 17 “Partnership for the Goals”	Inability to work; increase inequalities due to health conditions; ecosystem balance; global cooperation

From Table 2, it can be identified that the goals under this research are more strongly interconnected but additionally interconnected with SDG 15, SDG 9, SDG 10, SDG 5, SDG 1, SDG 17 and SDG 11. The absence of connections with SDG 2, SDG 6, SDG 7, SDG 14 and SDG 16 means no direct immediate implication for the analyzed SDGs in the context of this study.

### Green recovery: the way forward

The unusual situation created by the pandemic of COVID-19 has severe consequences for society, while the management of these consequences and policy responses will impact future developments. The pandemic is acting as a driving force for changes in main sectors and highlights the interdependence of sustainability issues embraced in the SDGs. Lock-down related effects such online education, forced home-office work, unemployment or decreased working hours, reduced mobility, etc., have enhanced social-structural injustice and inequality (SDG5) and (SDG10). Observations and analyses also indicate stronger dependencies between SDG4, SDG8, SDG3 and SDG13.

Nevertheless, the pandemic has presented a unique opportunity for change, as more people are aware of the urgency in which sustainability issues must be addressed and of the importance that balanced ecosystems have for health and wellbeing. There is also growing pressure for innovation (SDG9) and collaboration (SDG17). Therefore, it is important to seize the opportunity to rebuild and recover from the pandemic in a way that tackles sustainable development and builds resilience into communities.

Green recovery, although a long-term process, can be a response. The creation of green jobs via green stimulus must be accompanied by investment in training and digital capacities to create an equitable and inclusive green economy. In addition, it is important that governments create incentives for private sectors to invest in green innovation. Citizens’ opinions and behavior change can support sustainable policies and actions of public and private institutions. The interdependencies and synergies between the Goals highlighted during the pandemic can be considered during the recovery processes, i.e., financing cluster SDGs, which can increase their impact and efficacy.

The pandemic has shown the ability of governments to take dramatic action during times of crisis. In the long term, governments must collaborate and use international examples to learn about green recovery solutions. The UK government has proposed a Sustainable Recovery Alliance which would act as a forum for nations to discuss green recovery and coordinate recovery packages to maximize their impact. Multilateral institutions such as the UN and the IMF have encouraged their member states to fund solutions for urgent health and economic challenges, but success depends on the extent to which member countries cooperate.

The UN HLPF (2020) drew attention to the importance of global solidarity via multilateral institutions because the impacts of COVID-19 have been most devastating for the poorest and most vulnerable. This signifies a need for a strong commitment to accelerating policies that will facilitate the achievement of the SDGs to ensure that no countries are left behind in the global recovery effort [2]. Stronger alliances between nations are needed [76], and cooperation is needed to oversee resilience building in global supply chains and to support lower- and middle-income countries with fiscal and monetary policies.

In addition, climate change is a global issue that transcends political boundaries, so strengthening global cooperation is an integral part of achieving the SDGs. Furthermore, learning from current COVID-19-related experiences, decisions and policies can also lead to lessons learned for tackling the climate and environmental crisis the world is facing. The 2030 Agenda for Sustainable Development, which encompasses sustainability in all forms, can be a useful framework and guideline toward a sustainable future.

## Conclusion

Recent studies in the field on SDGs under COVID-19 primarily focus on effects on one selected SDG or one specific sector/thematic issue [77–81], or deal with the continuation of the SDGs [16, 82]. The research objective the article puts forward is to explore the way the pandemic impacts the SDGs, primarily their interconnections. The main focus of our study relates to the interconnectedness of SDGs and relies on goal to goals interconnection [49]. The specific interest is directed at better understanding the emerging effects on SDG interconnectedness through the new conditions under the influence of the COVID-19 pandemic and its management. We take those SDGs into consideration that are currently discussed as most affected.

Our qualitative approach based on expert FGDs proved capable of bringing up new results in an emerging field of research. In this regard, we shed light onto the contextualized SDGs interconnectedness under conditions of COVID-19. We present an overview of the identified interconnections between topics related to education, workforce and wellbeing, consumption and production, and climate change. Finally, based on our data analysis we extend the original focus of our analysis and depict SDG5 & SDG10 as critically affected by their interconnectedness not only under immediate influence of COVID-19 pandemic, but also by means and effects of the crisis management. The analysis can further be developed to expand the areas of focus and expand our understanding of how the SDGs are interconnected in such special times.

*Limitations of the study*

Given the early stage of research on the impact of the COVID-19 pandemic on the SDGs and possibly interconnected effects, the study encounters limitations, primarily related to the limited numbers of SDGs covered and experts involved in qualitative analysis.

A general evaluation of interaction between the SDGs is done at the goal level, not at the target or indicator level. These evaluations are done based on expert opinions not on SDGs data. Advanced analyses for interactions at the target or indicator level, based on data, can be the subject of another study. Different contexts could produce different results, as the SDG linkages are context dependent.

## Annex

See Table 3.

**Table 3:** Profiles of the focus group participants and facilitators and overview of the online sessions (June–October 2020)

Sessions/presenter	Description
Session 1 Giuseppe Scandone Leader of QG Enviro, Italy	Environmental education in the pandemic: a chance for rethinking QG Enviro, before the pandemic was strongly focused on international projects on environmental education and the SDG's. Youth Exchanges, etc. But lockdown and travel restrictions changed the situation. Is it really important for young people to travel around for short term projects? How can we correlate better the international work we do with the local communities in our region? If we start working more on online programs, what are the pros and cons of it? The discussion is still ongoing, some potential solutions are slowly crafted. What we all agree in QG Enviro is that the next future will see our organization developing at least another field or SDG (sustainable agriculture, tourism), in order to have a better resilience to unexpected events
Session 2 Dr. Stefan Cibian Executive Director Făgăraș Research Institute ICF Rumania	Global trends in international development with impact on global sustainability Departing from the transformation that the Sustainable Development Goals (SDGs) have brought with regard to the global efforts to foster development and enhance sustainability. The SDGs represent, in many ways, a departure from previous approaches to doing development, emphasizing the universality of assumed targets, a much wider thematic agenda, new central actors, and an important focus on finances. All of these are deeply affected by the COVID-19 crisis. During the discussion we will explore together the implications of the crisis on the SDGs and the macro-trends in development
Session 3 Edna Martinez Consultant in Sustainability Proactivo Sostenible—Mexico	Sustainable Production and Consumption as driver of the SDGs in the post COVID era From global to local and from waste to no trace, consumption will drive the agenda post COVID-19. Companies will boost the implementation of the agenda from their social corporate responsibility moving from traditional welfare to an essence where their contribution will respond to their core business. Institutions (public and private) are in the magnifying glass, there governances, social and environmental impact is being scrutinized by citizenship and consumers. Their role is being either applauded or taunted, this will make a significant difference in the implementation of the 2030 Agenda that could boost sustainable development
Session 4 Pourya Saleehi Urban planner, and sustainability and resilience expert who is also the Researcher Officer at ICLEI World Secretariat in Bonn	The impacts of COVID-19 on SDGs: "The critical aspects and potential consequences of COVID-19 for the achievement of Sustainable Development Goals (SDGs): Green Recovery as a way forward" Discussion about the green recovery as a way forward for bouncing back better from COVID-19. Exploring how COVID-19 offers an opportunity to accelerate towards achieving the SDGs as behavior changes have begun to shift towards green practices in some aspects of everyday life, and the importance of protecting ecosystems has been made highly apparent
Session 5 Filip Nelkovski Senior Project manager (UNDP) innovation, building resilient infrastructure North Macedonia	Taking advantage of the crisis towards building Sustainable cities and communities: "The impacts from pandemic on urban mobility and How COVID-19 helps in meeting SDGs for creation of car-free cities If we want to act and save the planet, to meet the SDG's on sustainable communities and see people moving freely not by car, a more revolutionary approach is needed. COVID-19 did a lot of problems in our lives. However, the pandemic also helped communities to start more direct approach in delivering mobility in the cities still governed by the cars. Reducing our dependence on petrol cars is not only good for personal health, but it creates a better future for all
Sessions 6 Prof. Dr. Bernd-Friedrich Voigt Professor of business psychology at FOM—Hochschule für Oekonomie & Management Münster/Germany	Title: COVID-19 induced technostress and its effects on SDGs 3 and SDG8 Recent developments under COVID-19 have brought radical changes for most of us—at work and at home, likewise. Our professional, our social and our private lives are more than ever forced into a digital environment. An enormous number of the working population, is reported as of feeling overly stressed by the radicalness of changes and the technological impetus. A currently running study among 1000 German workers allows the preliminary conclusion, that technostress and technostrain under the conditions of COVID-19 lockdown and extended home-office can be seen as substantial threats to SDGs 3 (Good Health And Well-Being) and 8 (Decent Work And Economic Growth). In addition to technologically induced stress, insufficient self-management competences and poor digital leadership must be seen as pivotal strain enhancers
Session 7 Dr. Kalterina Shulla Chair RCE middle Albania Associated Researcher/ZEF Centre for Development Research/University of Bonn	Discussion of the results of the individual sessions Evaluation of the findings from the six individual sessions and revaluation of how far the analyses represent the group discussions

Pproject: effects of COVID-19 on the Sustainable Development Goals (SDGs), funded by iac Berlin

## Data Availability

The datasets generated during and/or analysed during the current study are available from the corresponding author on reasonable request.
